# Supplementary material for: Immunoglobulin Superfamily Containing Leucine-Rich Repeat (ISLR) Serves as a Redox Sensor That Modulates Antioxidant Capacity by Suppressing Pyruvate Kinase Isozyme M2 Activity
Source: Cells. 2024 May 14;13(10):838. doi: 10.3390/cells13100838 (PMC11119796; doi:10.3390/cells13100838)
Supplement: Supplementary file 1 [file cells-13-00838-s001.zip › cells-2943193-supplementary.pdf]

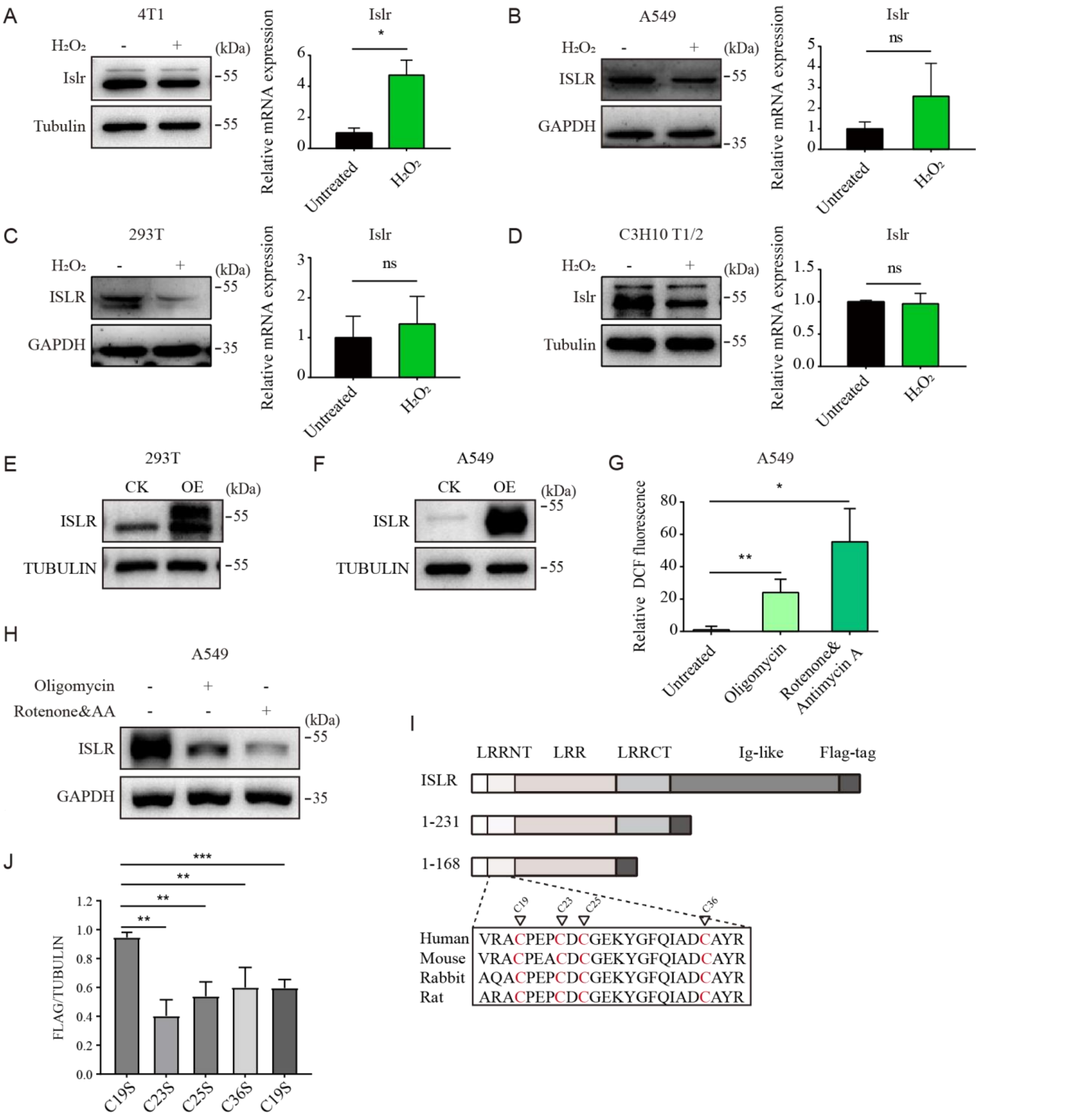

Fig. S1. ISLR is suppressed by oxidative stress. (A) 4T1 cells were treated with 2 μM H_2O_2 for 3 h, cells were analyzed with western blotting (left) or quantitative reverse- transcription polymerase chain reaction (qRT -PCR) (right), relative mRNA expression was normalized to GAPDH. (B) A549 cells were treated with 10 μM H_2O_2 for 3 h, cells were analyzed with western blotting (left) or qRT -PCR)(right), relative mRNA expression was normalized to GAPDH. (C) HEK293T cells were treated with 1 μM H_2O_2 for 3 h, cells were analyzed with western blotting (left) or qRT -PCR (right), relative mRNA expression was normalized to GAPDH. (D) C3H10 T1/2 cells were treated with 10 μM H_2O_2 for 3 h, cells were analyzed with western blotting (left) or qRT -PCR (right), relative mRNA expression was normalized to GAPDH. (E) HEK293T cells were transiently transfected with ISLR overexpression plasmid for 48 h. (F) A549 cells stably expressing ISLR were selected by G418. (G and H) A549 cells stably expressing ISLR were treated with 10 μM Oligomycin or Rotenone& Antimycin A

(AA) for 6 h, ROS levels were determined by fluorescence of oxidized DCFDA (G). ISLR level were determined by western blotting (H). (I) Schematic diagram of ISLR structure. LRR, leucine-rich repeat; LRRCT, Leucine rich repeat C-terminal domain. (J) Densitometric analysis of endogenous FLAG expression in Figure 1I, normalized to each untreated control. Data are means ± SEM (n⩾5). $^*P<0.05$； $^{**}P<0.01$; ns, not significant.

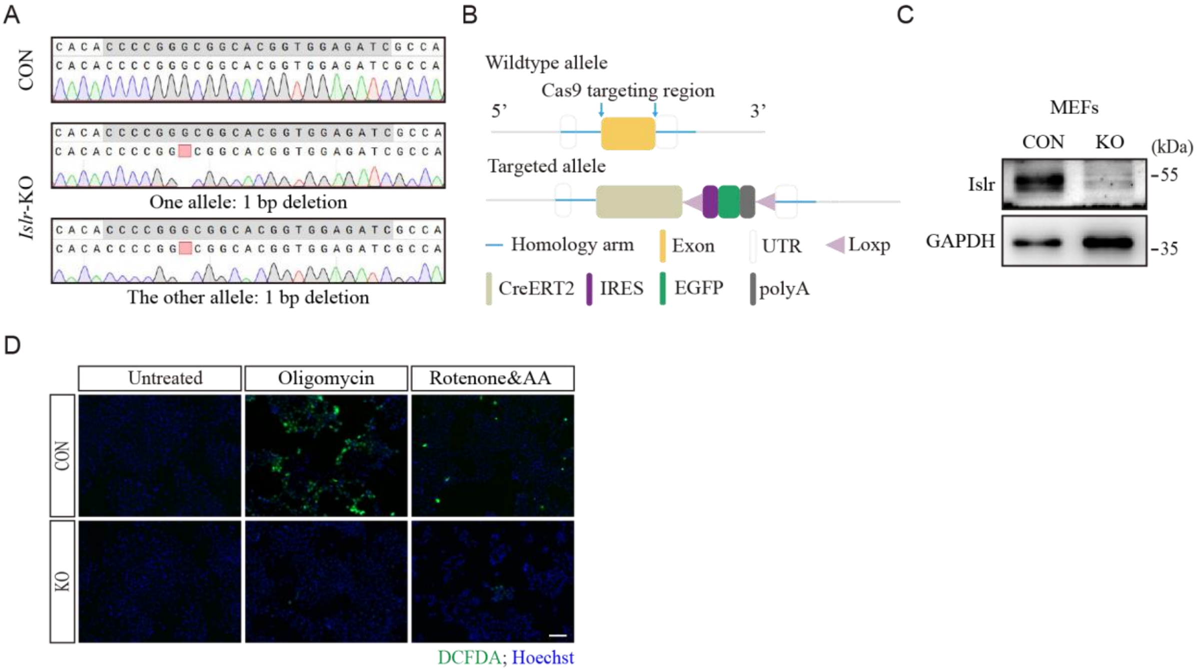

Fig. S2. ISLR decreases antioxidative capacity in response to oxidative stress. (A) Sequencing of wildtype and *Islr*-knockout 4T1 cells. (B) Schematic diagram of *Islr*-knockout mice. (C) Western blot analysis of ISLR in mouse embryonic fibroblasts (MEFs) which isolated from wildtype and *Islr*-knockout mice. (D) Representative pictures showing changes in ROS levels of 4T1 cells treated with 10 μM Oligomycin or Rotenone& Antimycin A for 6 h. Scale bar=100 μm.

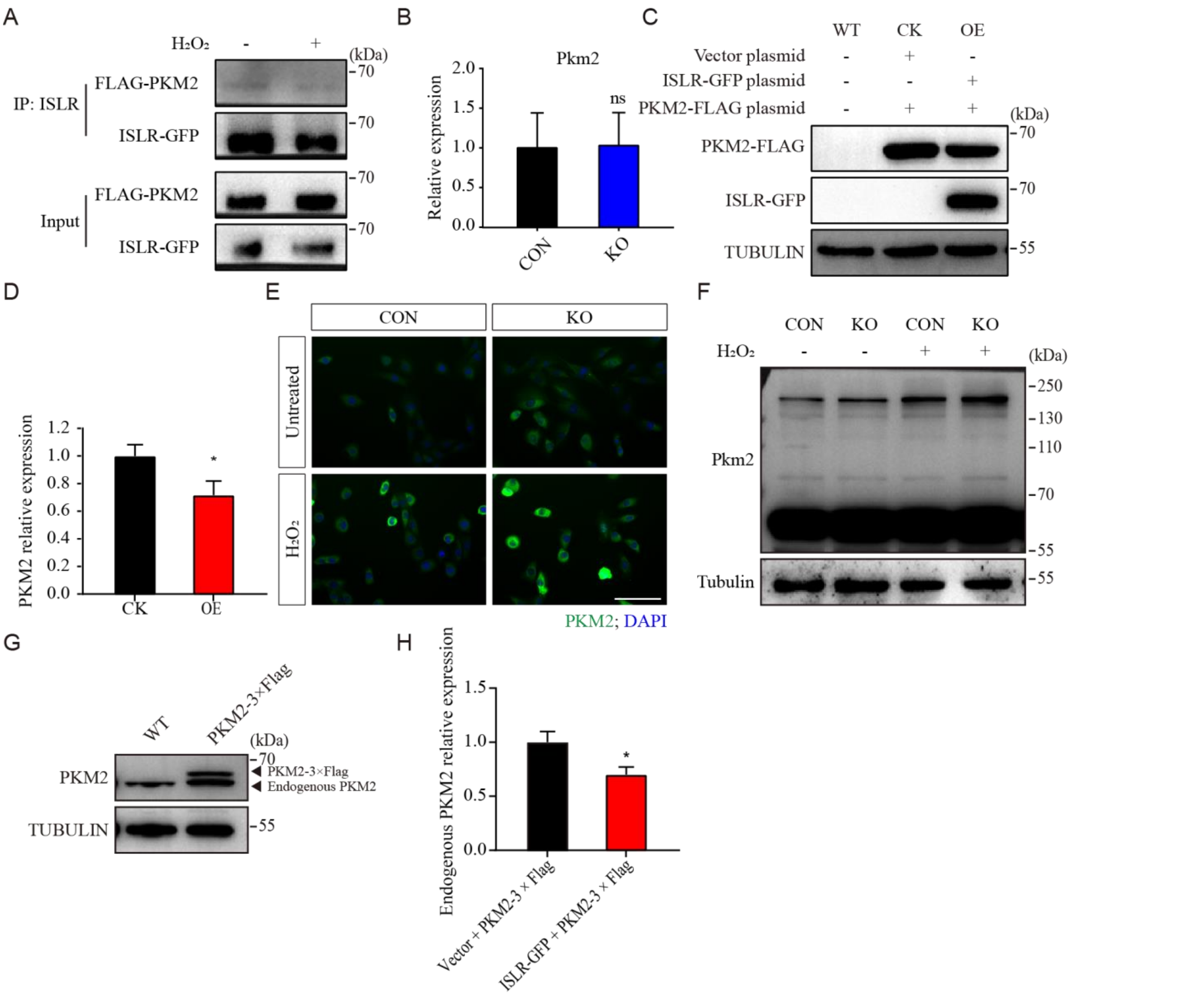

Fig. S3. Islr suppresses PKM2 expression. (A) Reciprocal co-immunoprecipitation analysis between Islr-GFP and PKM2-Flag in HEK293T cells cultured for 2 d, with or without 2 μM H_2O_2 treatment for 3h. ISLR antibody was used as the bait. (B) Control and *Islr*- knockout 4T1 cells were analyzed with qRT -PCR to test the expression of Pkm2. (C) Western blot analysis of ISLR and PKM2 in HEK293T cells which were transfected with ISLR overexpression plasmid for 1d, followed with transfection of PKM2-Flag plasmid for 2 d. (D) Densitometric analysis of PKM2 level in (C), normalized to CK group. (E) PKM2 level in C2C12 cells treated with or without H_2O_2 were analyzed using IF. (F) C2C12 cells stably expressing ISLR were treated with 1 μM H_2O_2 for 3 h, PKM2 dimer and tetramer formation analyzed in DSS cross- linked C2C12 cells. (G) HEK293 cells transient expressing PKM2-3×Flag plasmid were analyzed using western blotting. (H) Densitometric analysis of endogenous PKM2 expression in Figure 4G, normalized to Vector +PKM2-3×Flag group. Data are means ± SEM (n⩾5). *$P < 0.05$； **$P < 0.01$; ns, not significant.

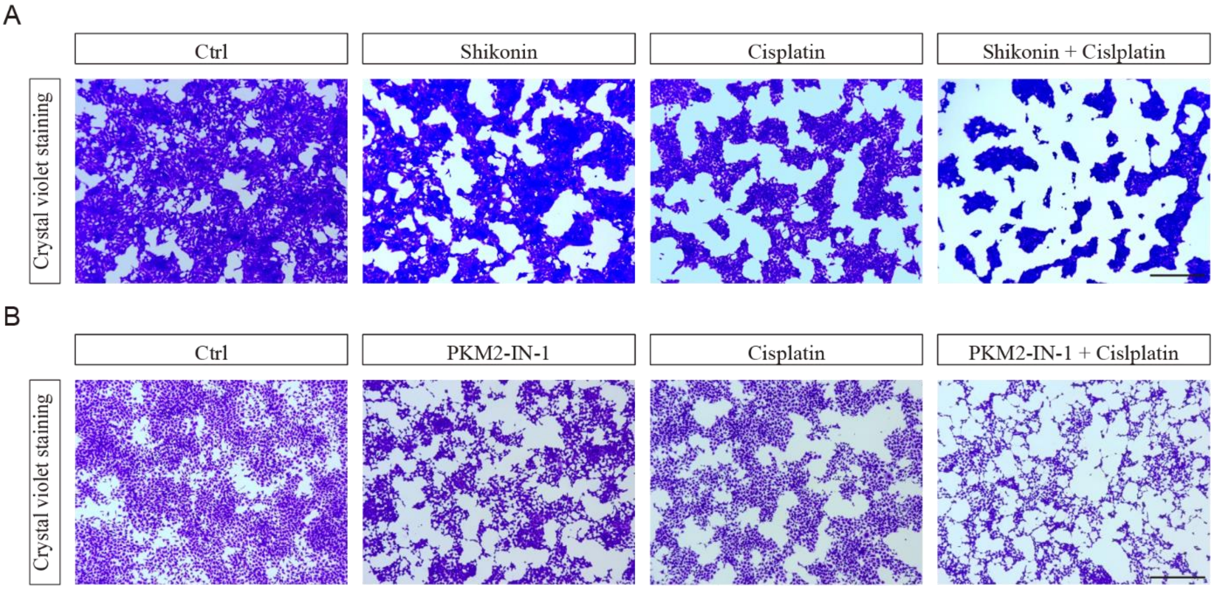

Fig. S4. PKM2 inhibition sensitizes tumor cells to cisplatin. (A) Crystal violet staining of 4T1 cells which were treated with 1 μM Shikonin/ 1 μg/ml Cisplatin or both reagents for 9 h. bar=100 μm. (B) Crystal violet staining of 4T1 cells which were treated with 10 μM PKM2-IN-1/ 1 μg/ml Cisplatin or both reagents for 9 h. bar=100 μm.
